# Corrigendum to “Transcriptomic data of Bevacizumab-adapted colorectal adenocarcinoma cells HCT-116” [Data In Brief, Volume 48, (Available online 17 March 2023) 1-9/Article 109069]

**DOI:** 10.1016/j.dib.2023.109343

**Published:** 2023-06-24

**Authors:** Cesare Sala, Tiziano Lottini, Elena Lastraioli, Federico Alessandro Ruffinatti, Luca Visentin, Annarosa Arcangeli

**Affiliations:** a Department of Experimental and Clinical Medicine, University of Florence, Viale GB Morgagni 50, 50134 Florence, Italy; b Complex Dynamics Study Centre (CSDC), University of Florence, 50100 Florence, Italy; c Department of Life Sciences and Systems Biology, University of Torino, Via Accademia Albertina 13, 10123 Torino, Italy

The authors regret **Cesare Sala^a^,^1^, Tiziano Lottini,^1^, Elena Lastraioli^a^,^b^, Federico Alessandro Ruffinatti^c^,Luca Visentin ^c^, Annarosa Arcangeli^a^,^b^,^∗^**

The authors would like to apologise for any inconvenience caused.

